# Accurate few-shot object counting with Hough matching feature enhancement

**DOI:** 10.3389/fncom.2023.1145219

**Published:** 2023-03-30

**Authors:** Zhiquan He, Donghong Zheng, Hengyou Wang

**Affiliations:** ^1^Guangdong Key Laboratory of Intelligent Information Processing, Shenzhen, China; ^2^Guangdong Multimedia Information Service Engineering Technology Research Center, Shenzhen University, Shenzhen, China; ^3^School of Science, Beijing University of Civil Engineering and Architecture, Beijing, China

**Keywords:** few-shot, object counting, Hough matching, feature enhancement, exemplar feature aggregation, self-attention

## Abstract

**Introduction:**

Given some exemplars, few-shot object counting aims to count the corresponding class objects in query images. However, when there are many target objects or background interference in the query image, some target objects may have occlusion and overlap, which causes a decrease in counting accuracy.

**Methods:**

To overcome the problem, we propose a novel Hough matching feature enhancement network. First, we extract the image feature with a fixed convolutional network and refine it through local self-attention. And we design an exemplar feature aggregation module to enhance the commonality of the exemplar feature. Then, we build a Hough space to vote for candidate object regions. The Hough matching outputs reliable similarity maps between exemplars and the query image. Finally, we augment the query feature with exemplar features according to the similarity maps, and we use a cascade structure to further enhance the query feature.

**Results:**

Experiment results on FSC-147 show that our network performs best compared to the existing methods, and the mean absolute counting error on the test set improves from 14.32 to 12.74.

**Discussion:**

Ablation experiments demonstrate that Hough matching helps to achieve more accurate counting compared with previous matching methods.

## 1. Introduction

Object counting (Zhang et al., 2017), which aims to count the number of objects of interest in images or videos, has become a research hotspot in computer vision. Most existing object counting methods require the target object of the test data to appear in the training stage. In class-specific object counting, such as people (Liu et al., 2021; Zhang et al., 2021), car (Hsieh et al., 2017), or cell counting (Xue et al., 2016), the classes in both the training and test sets are unique and identical. Therefore, a well-learned model can only handle a certain category that has been covered in the training set and is unable to count other categories, which limits the application of the counting model. The cost of the manual mark is expensive, and the number of available samples is rare in some classes.

Given some images, the human can easily generalize novel concepts and search the same class objects in query images, even if the objects vary in shape, illumination, scale, and so on. Inspired by the human's ability to quickly generalize new concepts, few-shot object counting (FSC) (Lu et al., 2018), which can count the novel classes that are not present in the training stage, is proposed to solve the generalization obstacle. Specifically, when the user specifies random exemplars, FSC only counts the corresponding sample class objects in query images. During training, only the base classes (seen) are used, and inference is performed on the novel classes (unseen). In this way, few-shot counting can apply the experience learned in the base classes to the novel classes.

FSC is a challenging problem that still needs further study. Generally, existing methods works in two stages: feature extracting and matching. They extract the features of the query image and samples, and similarity-matching results are used as an essential basis to infer the object counts of the query image. Existing methods most use convolutional neural networks (CNN) as feature extractor and design their matching network. GMN (Lu et al., 2018) concatenate the query feature and features on the channel dimension, but the similarity maps is ignored. FamNet (Ranjan et al., 2021) maps the exemplars and query images to the similarity space, and generate a density map from it. However, only using the similarity map to infer the density map is not accurate, and the query feature is not fully utilized. BMNet+ concatenate the similarity map and the query feature into the counter, while the border of the density map is blurred when the objects are dense. SAFECount (You et al., 2023) improves the counting accuracy by means of feature enhancement, but the effect is not ideal when the object is occluded.

With the development of the deep neural network, CNN have made impressive progress in robust feature representation for establishing correspondences. Feature matching usually adopts the result of the convolution of query features and sample features. However, it remains challenging for correspondent matching in the presence of intra-class variations, which refers to the variations of the different instances of the same class. And spatial matching with a geometric constraint is still effective when facing blur, occlusion, illumination changes, and so on. It can reduce the number of uncertain candidate regions with reliable inference and is adopted by many methods (Cho et al., 2015; Han, 2017; Min et al., 2020).

Hough transform has long been used as geometric verification for rigid object matching (Hough, 1962). Ballard (1981) summarizes the main idea of the Hough transform as voting in parameter space with R-table, whereby the detection of arbitrary objects is achieved. Hough matching has been widely applied in various tasks such as object detection (Gall and Lempitsky, 2013; Milletari et al., 2017), 3D vision (Knopp et al., 2011), and pose estimation (Kehl et al., 2016). Recent works (Han, 2017; Min et al., 2019) have developed the idea of Hough transform to conduct non-rigid matching in point-to-point semantic correspondence, but object-to-object matching remains to be explored.

In this work, we propose a Hough matching feature enhancement network for FSC, which learns a flexible non-rigid matching kernel to increase the reliability of matching results. We use a local self-attention module to improve the quality of the query feature tensor. Exemplar feature aggregation is applied to enhance the commonality of exemplar feature tensors. The Hough matching creates the original similarity space of candidate matches and evaluates them in a convolutional manner. The convolutional way makes the output pay attention to each position with its surrounding context and equivariant to translation. To further pull close the objects that are similar to samples, the sample features would be fused into query features according to the weight of the Hough matching result. And we use a cascade structure to connect the same network in series, which can pull the samples and targets closer and push the samples and background away.

Our contribution can be summarized as follows:

- We introduce the local self-attention module to optimize the semantics of feature vectors by incorporating contextual information.
- We extract the common feature of all exemplars and add it to each exemplar to enhance the commonality of samples.
- We propose an object-to-object Hough matching module that votes in the Hough space, which optimizes the similarity map in the form of convolutions.

This article is presented as follows. Section 2 introduces the related work. The architecture of the Hough matching feature enhancement network (HMFENet) is presented in Section 3. Section 4 offers the evaluation results compared with previous methods and gives ablation studies. Finally, a brief conclusion is made in Section 5.

## 2. Related work

### 2.1. Class-specific object counting

Class-specific object counting only counts for specific categories, such as vehicles (Hsieh et al., 2017), people (Liu et al., 2021; Song et al., 2021; Zhang et al., 2021), and animals (Arteta et al., 2016). And crowd counting is closely related to human society and has a wide range of uses in many fields, so this counting category has been widely studied. According to the accumulated methods, object counting can mainly be divided into detection-based counting and regression-based counting. The former relies on the target detector to obtain object location through target detection and can count the number of target objects at the same time. In recent years, target detection algorithms have developed rapidly. Algorithms such as YOLO (Redmon et al., 2016), RetinaNet (Hsieh et al., 2017), and CenterNet (Duan et al., 2019) have continuously improved the accuracy of target classification and positioning. However, target detection is not specially designed for the counting field. It needs to train detectors for different types of objects, and the training needs far more annotation information than the latter. In addition, its performance is not satisfactory when the objects are dense, occluded, overlapped, and so on.

Regression-based counting (Ma et al., 2019) learns maps from extracted image features to per-pixel density values based on ground-truth density maps. It only needs a small amount of annotation information during training. When annotating the dataset, it only needs to mark 1 in the center of each target object and use a Gaussian kernel to convolve the counting map to generate a density map. This type of annotation is more efficient and less labor-intensive than rectangular box annotation. Most methods pay attention to designing effective network architectures (Zhang et al., 2016), multi-scale framework (Zeng et al., 2017), or self-attention (Jiang et al., 2020). Compared with few-shot counting, class-specific object counting lacks the feature matching stage.

### 2.2. Few-shot object counting

The purpose of few-shot object counting (FSC) is to bridge the knowledge gap between the base class and the novel class and strengthen the generalization performance of the counting models. Given K exemplars, the model must find and count objects of the same category as the exemplars in the query image. Thus, the task is also called K-shot FSC. With the development of few-shot learning, few-shot object detection (FSOD) received attention, which is the extension of FSC. It can detect the locations of the novel class objects, and specify its category when given several objects of different novel classes. However, FSC still has an advantage over FSOD in terms of data annotation costs.

Lu et al. (2018) first proposes a generic counting model for FSC, which uses a sharing convolutional neural network to extract the query and exemplar feature maps and concatenate them to regress the object count. Recent works consider making full use of similarity maps. Yang et al. (2021) designs a multi-scale matching network and gets similarity maps by using the exemplar feature map as kernel to convolve the query feature map. Ranjan et al. (2021) also model the similarity map by means of convolution and send the similarity map to the regress head. However, the query feature is not fully utilized, and the boundary of the density map is fuzzy. Shi et al. (2022) uses self-attention to narrow the distance between the samples and the target objects and then concatenate the similarity map to regress head. But when the objects are dense and the light is dim, the boundary of the density map is not clear, and the accuracy of the count value is low. You et al. (2023) adopted feature enhancement guided by similarity maps, which refines the query feature tensor and then regress it to obtain a density map. When the objects are occluded and overlapped, the effect will also decrease.

However, these methods of obtaining similarity maps through convolution need to be improved, especially in the face of severe deformation and occlusion. Inspired by the successful application of point-to-point Hough matching (Min et al., 2020), we propose object-to-object Hough matching in FSC. In this work, our Hough matching module is an effective method when facing non-rigid deformation.

## 3. Methods

**Figure 1:**
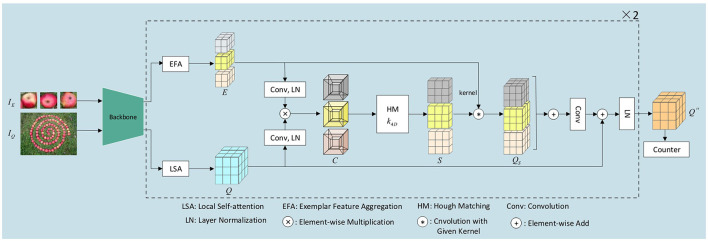
Illustration of the Hough matching feature enhancement network.

In this section, we introduce our network HMFENet for FSC, which use Hough matching to get the similarity map between the exemplars *I*_*E*_ and the query image *I*_*Q*_. First of all, we use CNN as our backbone to extract the image feature and refine it through local self-attention (LSA). And we design an exemplar feature aggregation (EFA) module to enhance the commonality of the exemplar feature. Second, the learnable Hough matching (HM) module outputs reliable similarity maps. Then, we augment the query feature with exemplar features according to the similarity maps, and use a cascade structure to further enhance the query feature. Finally, we send the refined query tensor to the counter module, which outputs the density map. We can simply sum the density map to get the final number of objects. Figure 1 illustrates our overall architecture.

### 3.1. Local self-attention

Given a feature tensor of query image *X*, the local self-attention (LSA) module optimizes each feature point *X*_*ij*_ ∈ ℝ based on the context information. We extract a local region feature tensor with spatial extend *r* × *r* surrounding *X*_*ij*_. As common self-attention framework (Ramachandran et al., 2019; Zhao et al., 2020; Vaswani et al., 2021), our local self-attention conducts on queries (q), keys (k), and values (v) with an input feature map *X*, and output a optimized version $\tilde{X}$, which is the same shape as *X*.

**Figure 2:**
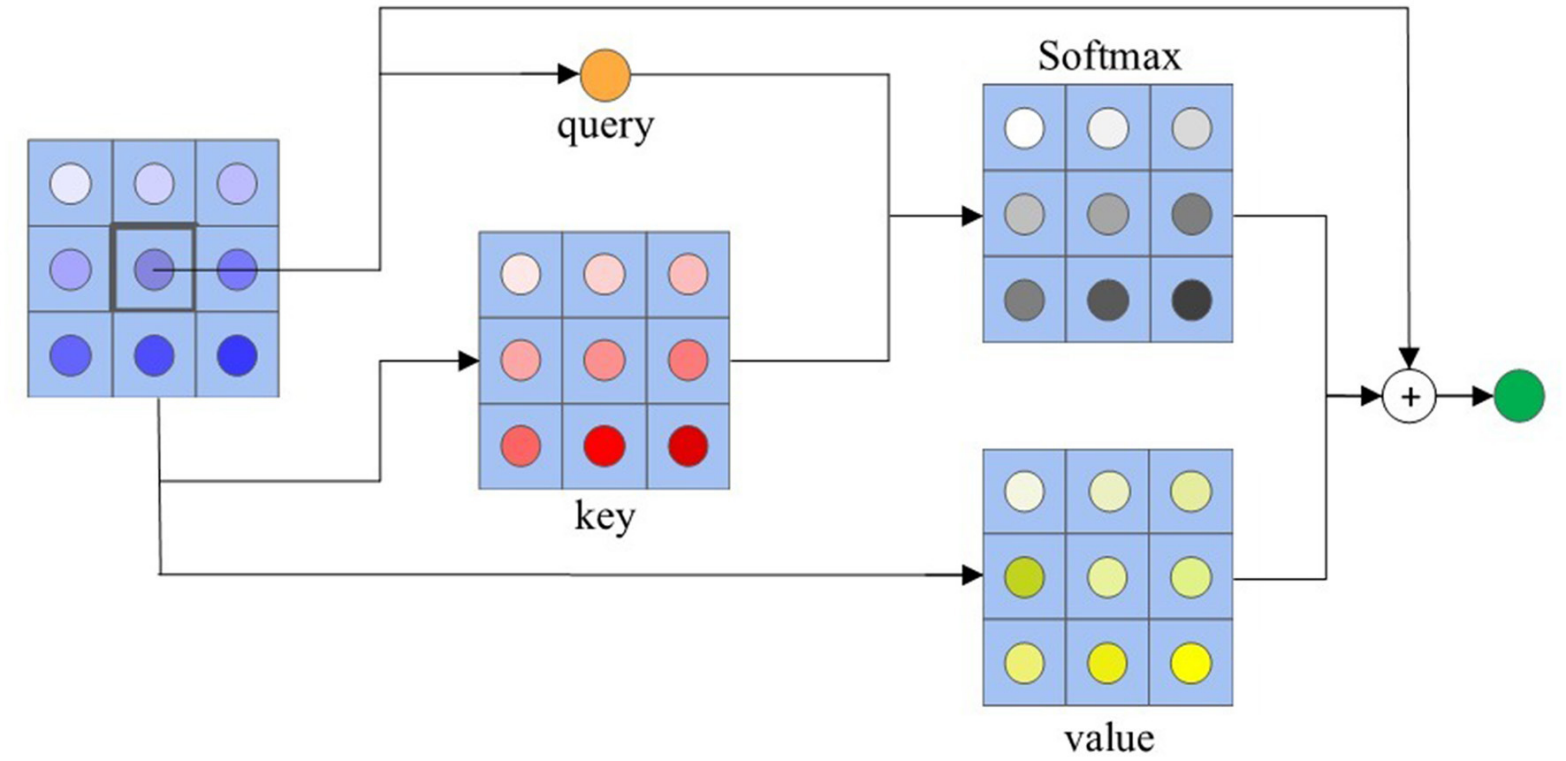
An example of local self-attention with *r* = 3.

Let *C, H*_*q*_, and *W*_*q*_ represent the channel, height, width of query tensor, respectively. For any position *ij*, we select its *r*×*r* neighborhood to operate the self-attention. We collect the feature vectors of the neighborhood locations in *ij*, and neighborhood feature tensor $X^{\prime }\in {\mathbb{R}}^{C\times H_{q}\times W_{q}\times r\times r}$ can be obtained. Then, we feed the feature map *X* into the query transformation function $F_{q}$, and feed the neighborhood feature tensor *X*′ into the key and value transformation functions $F_{k}\text{,}F_{v}$, as shown in Figure 2. Our transformation functions are implemented with independent 1 × 1 convolutions followed by ReLU activations. The local self-attention in each feature point can be described as the following equation:


(1) $$\begin{array}{l} Q_{ij}=X_{ij}+\mathrm{Conv}\left(F_{v}\left(X_{ij}^{\prime }\right)\mathrm{SoftMax}{\left(F_{q}{\left(X_{ij}\right)}^{\text{T}}F_{k}\left(X_{ij}^{\prime }\right)\right)}^{\text{T}}\right). \end{array}$$

where $X_{ij}^{\prime }$ is the neighborhood feature tensor in the location *ij* of input feature tensor *X*, $F_{q}\left(X_{ij}\right)\in {\mathbb{R}}^{C^{\prime }}$ is *ij*^*th*^ query, $F_{k}\left(X_{ij}^{\prime }\right)\in {\mathbb{R}}^{C^{\prime }\times r\times r}$ and $F_{v}\left(X_{ij}^{\prime }\right)\in {\mathbb{R}}^{C^{\prime }\times r\times r}$ is *ij*^*th*^ key and value, and Conv are conducted by 1 × 1 convolutions.

### 3.2. Exemplar feature aggregation

In previous methods (Shi et al., 2022; You et al., 2023), the exemplar features from multiple shots are usually used to calculate correlation tensor independently, and the commonality of multiple exemplar features is underutilized. Therefore, we build an exemplar feature aggregation (EFA) module, which leverages the features from every exemplar to enhance the commonality.

Let *ϕ* (*s*^*i*^), *i* = 1, ⋯ , *k* represent the k-shot exemplar features, and exemplar feature aggregation can be expressed as a weighted average of the features:


(2) $$\begin{array}{l} E_{i}=\phi \left(s^{i}\right)+\overset{k}{\underset{j=1}{\sum }}\left(\phi \left(s^{j}\right)⊗C^{j}\right), \end{array}$$

Where ⊗ is element-wise multiplication and *C*^*j*^ is correspondent coefficient. *E*_*i*_ is the *i*^*th*^ optimized exemplar feature and we use ***E*** to represent all optimized exemplar features.


(3) $$\begin{array}{l} C^{j}=\mathrm{SoftMax}\left(\mathrm{MLP}\left(f^{j}\right)\right), \end{array}$$

Where ***f***^*j*^ is the output of exemplar commonality extractor:


(4) $$\begin{array}{l} f^{j}=ℱ(\mathrm{Conv}\left(\phi \left(s^{j}\right)\right),\frac{1}{k}\sum_{i=1}^{k}\mathrm{Conv}\left(\phi \left(s^{i}\right)\right), \end{array}$$

and the function F is represented as followed:


(5) $$\begin{array}{l} F\left(\text{A},\text{B}\right)=\mathrm{Conv}\left(\mathrm{Cat}\left(\text{A},\text{B}\right)\right)+\mathrm{Cat}\left(\mathrm{Conv}\left(\text{A}\right),\mathrm{Conv}\left(\text{B}\right)\right) \end{array}$$

Where Conv is convolution, and Cat is the channel concatenation.

After that, we perform Hough matching and feature enhancement.

### 3.3. Hough matching

The Hough transform is a powerful method for geometric object detection, which votes for candidate target objects in a parameter space, also called Hough space. The traditional Hough matching builds reliable correspondences by geometric voting from candidate matching regions, and then target objects are detected by identifying the locations of local maxima in Hough space. However, traditional Hough matching is weak to background noise and gets unsatisfied performance in the face of non-rigid matching. So, we propose a learnable Hough matching module to deal with the non-rigid object-to-object matching problem.

To alleviate the computational burden, we use max-pooling to reflect the importance of exemplar feature information. The exemplar features' size reduces to 3 × 3 in height and width. Given the query and exemplar feature tensors, we use shared convolution kernels and layer normalization to make the exemplar feature and the query feature subject to the same distribution. We construct the Hough space of geometric transformation by computing all possible 4D correlation tensors:


(6) $$\begin{array}{l} C_{i}=\mathrm{RELU}\left(E_{i}\cdot Q\right), \end{array}$$

Where $C_{i}\in {\mathbb{R}}^{H_{e}\times W_{e}\times H_{q}\times W_{q}}$. *H*_*e*_ and *W*_*e*_ are the height and width of the exemplar feature after max-pooling. RELU can simply suppress negative matching to zero.

We use a convolutional Hough matching kernel to accumulate the matching votes.


(7) $$\begin{array}{l} v_{i}\left(\text{h}\right)=b+\underset{\left(\text{x},{\text{x}}^{\prime }\right)\in E_{i}\times Q}{\sum }C_{i}\left(\text{x},{\text{x}}^{\prime }\right)kernel\left(\left||\left({\text{x}}^{\prime }-\text{x}\right)-\text{h}|\right|\right), \end{array}$$

Where ||·|| is a distance function that computes the distances with the center of kernel, **h** is an offset in Hough space, and b is a learnable bias.

We merge the dimensions *H*_*e*_ and *W*_*e*_ of correlation tensors into one dimension, and then the kernel is implemented with 3D convolution over the correlation tensors. We design the kernel size as 3. The kernel computes the similarity maps between **x**′−**x**, observed matching offset, and the given offset **h** in the Hough space. It is hoped to learn a voting weight for each candidate match based on the offset caused by the matching result. We consider that the matching results are more correlated with the distance to the kernel center and less correlated with the position direction of the parameters. Therefore, we use a position-sensitive isotropic kernel to share the training parameters according to the position direction.

We use the Hough matching results of the exemplar central feature points as object matching map $S_{c}\in {\mathbb{R}}^{k\times H_{q}\times W_{q}}$ and normalize it as follows:


(8) $$\begin{array}{l} S=\frac{\exp\left(S_{c}\right)}{\max\left(\exp\left(S_{c}\right),\dim=(2,3)\right)}⊗\frac{\exp\left(S_{c}\right)}{\text{sum}\left(\exp\left(S_{c}\right),\dim=1\right)}\;\\ \;\in {\mathbb{R}}^{k\times H_{q}\times W_{q}}. \end{array}$$

### 3.4. Feature enhancement

Recall the prior work by Ranjan et al. (2021). Although the similarity map reflects the matching confidence, its information in representing the target objects in image is less than the combination of query feature tensor and similarity map. We introduce the feature enhancement module and the cascade structure to fully use the similarity map ***S***, query feature tensor, and exemplar feature tensors.

Specifically, guided by the weight of the corresponding position of the similarity map, we integrate the exemplar features into the query tensor. In this way, the model will focus on areas similar to the exemplar in the query image. The similarity weight feature aggregation is implemented with convolution as follows:


(9) $$\begin{array}{l} Q^{\prime }=\overset{k}{\underset{i=1}{\sum }}\mathrm{Conv}\left(S,\text{kernel}=E_{i}\right)\in {\mathbb{R}}^{C\times H_{q}\times W_{q}}, \end{array}$$

Then, ***Q***′ goes through two convolution layers and is added to the query tensor ***Q*** in the form of residuals. Finally, we apply layer normalization to the output.


(10) $$\begin{array}{l} Q^{\prime \prime }=\text{layernorm}\left(Q+\mathrm{Conv}\left(Q^{\prime }\right)\right)\in {\mathbb{R}}^{C\times H_{q}\times W_{q}}. \end{array}$$

### 3.5. Cascade and counter

The final output ***Q***^′′^ is the same shape as the input query tensor. Thus, we can use the cascade structure to stack the same module. Specifically, we can replace ***Q*** with ***Q***^′′^ and concatenate an identical network structure except for the backbone. If the exemplar images are from the query image, we extract the exemplar features from ***Q***^′′^, otherwise use the original exemplar feature tensors. In this part, we cascade two times for further feature enhancement.

Following prior works (Lu et al., 2018; Shi et al., 2022; You et al., 2023), the counter is composed of several convolution layers and bilinear upsampling layers. The composition of counter is presented in Table 1, and each convolution layer is activated with the Leaky ReLU function. When the number of channels is reduced to 1, the height and width of the tensor are also restored to the resized image size, and the final output is the density map. We only need to sum the density map, and the value of counting can be obtained.

**Table 1:** Composition of counter.

**Layer**	**Kernel**	**In**	**Out**	**Followed by**
Conv	3 × 3	256	64	2 × Upsampling
Conv	3 × 3	64	32	2 × Upsampling
Conv	1 × 1	32	1	-

### 3.6. Loss function

The loss function is an essential part of deep learning. Most datasets use the center positions of target objects as annotations. It is difficult to obtain the position directly. Previous method (Shi et al., 2022; You et al., 2023) use adaptive Gaussian kernel to generate the ground-truth density map, but it is difficult to solve the object distortion caused by perspective effect. Here, we use a gaussian smoothing with a fixed size of 16 and a standard deviation of 3.5 to generate the ground-truth density map ***D***_*gt*_. Following previous methods (Lu et al., 2018; Shi et al., 2022; You et al., 2023), we use the mean squared error (MSE) loss function.


(11) $$\begin{array}{l} L=\frac{1}{H\times W}||D_{pr}-D_{gt}|{|}_{2}^{2}. \end{array}$$

Where *H* and *W* represent the height/width of the query image after resizing, respectively, and ***D_pr_*** is the density map output by the model.

## 4. Experiment

Here, we conduct the experiments on public datasets and validate the advantage of our model compared with other methods. Then, we analyze the influence of our modules. Although our model is designed for FSC, we also show the generality of our model on the class-specific dataset, a car counting dataset.

### 4.1. Implement detail

We use ResNet-18 (He et al., 2016) that pre-trained on ImageNet (Deng et al., 2009) as our backbone, which is also called feature extractor. The parameters of the backbone are fixed and do not participate in training. Given a query image, we resize the image size to 512 × 512 with bilinear interpolation. The outputs of the first three residual blocks of ResNet-18 are adjusted to the same size, and the shape of query tensor *C* × *H*_*q*_ × *W*_*q*_ is set as 256 × 128 × 128. We get the exemplar feature from the query feature when exemplar images are in the query image. And then, we resize the size of the exemplar tensor to 256 × 3 × 3 with max-pooling.

In local self-attention module, we set the neighborhood area *r* as 3, and the *C*′ is 1,024. The Conv in equation 1 is conducted by 1 × 1 convolutions, and the number of channels drop from 1,024 to 256. In EFA module, the input and output of the number of convolution channels are both 256. In feature enhancement module, the two convolution layers are performed by 3 × 3 convolutions. The channel first go up to 1,024 and go down to 256 in the second convolution layer.

We adopt Adam (Kingma and Ba, 2014) as our optimizer, and the model is trained for 150 epochs with an initial learning rate of 2*e* − 5. The learning rate drops to 5*e* − 6 at the 80th epoch. Our model occupies about 11G on NVIDIA GeForce RTX 2080Ti for training. In addition, we use gamma transformation and horizontal flip during the training stage to realize data augmentation.

### 4.2. Dataset and metric

The FSC-147 (Ranjan et al., 2021) dataset is the first and only large-scale dataset for few-shot counting. It contains 6,135 images from 147 classes, and each image has randomly selected three exemplars annotated by the bounding box to show the target objects. There are different cross-validation methods, such as k-fold cross-validation and jackknife test, which are generally used to develop deep learning model (Arif et al., 2018, 2020, 2021; Ge et al., 2021, 2022a,b; Sikander et al., 2022). According to the division method of the original dataset (Ranjan et al., 2021), we divide the dataset into training set, validation set, and test set. It should be noted that to validate the generality of FSC, the classes in training, validation, and test sets have no intersection. The training set has 89 categories, while the validation set and test set both own disjoint 29 categories, and the average of target objects is 59.

Mean Absolute Error (MAE) and Root Mean Squared Error (RMSE) are two standard metrics adopted by most counting methods, and we use them to evaluate the performance of our network.


(12) $$\begin{array}{l} \text{MAE}=\frac{1}{N_{i}}\overset{N_{i}}{\underset{i=1}{\sum }}|C_{i}-C_{i}^{gt}|, \end{array}$$

(13) $$\begin{array}{l} \text{RMSE}=\sqrt{\frac{1}{N_{i}}\overset{N_{i}}{\underset{i=1}{\sum }}{\left(C_{i}-C_{i}^{gt}\right)}^{2}}. \end{array}$$

Where *N*_*i*_ is the number of the query image. ***C_i_*** and $C_{i}^{gt}$ is the predicted and ground-truth count value of the *i*^*th*^ query image.

### 4.3. Quantitative results

**Figure 3:**
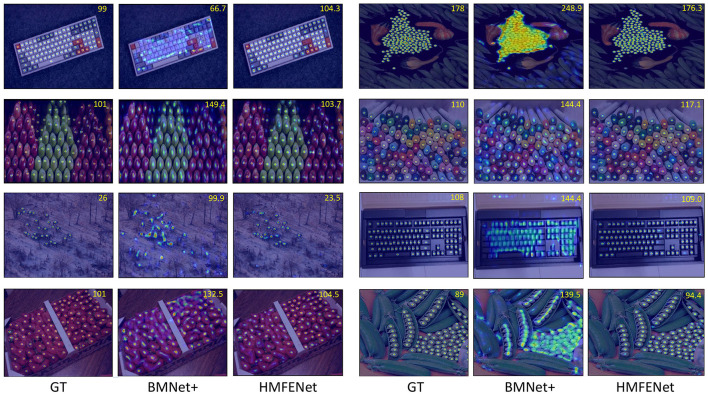
The visualization results on the FSC-147 dataset. From left to right, we sequentially place the visualization results of ground-truth density maps (GT), the predicted density maps output by BMNet+ (Shi et al., 2022), and the predicted density maps of our network HMFENet.

In this section, we evaluate our HMFENet model on the FSC-147 and compare it with other existing methods. As shown in Table 2, our results exceed the baseline SAFEcount and reach a new state-of-the-art. Our method outperforms SAFEcount by 2.18 MAE and 2.3 RMSE on the validation set. It also excels the SAFEcount by 1.58 MAE and 0.91 RMSE on the test set, which has 14.3% and 11.1% improvement on two MAE metrics, respectively. Figure 3 shows the qualitative results on the FSC-147 dataset.

**Table 2:** Results on FSC-147 dataset.

**Methods**	**Backbone**	**Val MAE**	**Val RMSE**	**Test MAE**	**Test RMSE**
FR (Kang et al., 2019)	YOLOv2	45.45	112.53	41.64	141.04
FSOD (Fan et al., 2020)	ResNet50	36.36	115.00	32.53	140.65
GMN (Lu et al., 2018)	ResNet50	29.66	89.81	26.52	124.57
MAML (Finn et al., 2017)	ResNet50	25.54	79.44	24.90	112.68
FamNet (Ranjan et al., 2021)	ResNet50	24.32	70.94	22.56	101.54
FamNet+ (Ranjan et al., 2021)	ResNet50	23.75	69.07	22.08	99.54
CFOCNet (Yang et al., 2021)	ResNet50	21.19	61.41	22.10	112.71
BMNet+ (Shi et al., 2022)	ResNet50	15.74	58.53	14.62	91.83
SAFECount (You et al., 2023)	ResNet18	15.28	47.20	14.32	85.54
HMFENet (our)	ResNet18	13.10	44.90	12.74	84.63

### 4.4. Ablation study

To fully prove the effectiveness of our module, we conduct thorough ablation studies, as shown in Table 3. In ablation studies without Hough matching, we use convolution between query and exemplar tensors to build similarity maps. We can make the following arguments. The local self-attention effectively (LSA) aggregates the neighborhood information of feature vectors and strengthens the semantic information of the query tensor. EFA enhances the commonality of multiple exemplars and is helped to the improvement of model performance. The cascade structure is helpful for feature enhancement. Hough matching builds a reliable matching map, which makes a significant improvement.

**Table 3:** Ablation study on FSC-147 dataset.

**No**.	**LSA**	**EFA**	**Cascade**	**HM**	**Val MAE**	**Val RMSE**	**Test MAE**	**Test RMSE**
1	✗	✗	✗	✗	16.57	54.38	16.43	95.71
2	✓	✗	✗	✗	16.04	54.26	15.71	94.46
3	✓	✓	✗	✗	15.42	53.18	15.10	91.27
4	✓	✓	✓	✗	14.10	48.58	14.04	89.67
5	✓	✓	✓	✓	13.10	44.90	12.74	84.63

### 4.5. Experiment on class-specific counting

CARPK (Hsieh et al., 2017) is a class-specific car counting dataset, which marks all target objects with the bounding box. The dataset owns 1,448 images of parking cars with a bird view.

Our method aims at the problem of FSC, but to verify the model's generality, we also carry out experiments in class-specific counting. Our model is first pre-trained on FSC-147 and then fine-tuned on the CARPK dataset. We randomly selected five exemplars in the query image in the fine-tuning stage. As shown in Table 4, our model is still better than theirs compared with few-shot object detection and other FSC methods.

**Table 4:** Results on CARPK dataset.

**Methods**	**Type**	**Method**	**MAE**	**RMSE**
YOLO (Redmon et al., 2016)	Detection	Generic	48.89	57.55
Faster-RCNN (Ren et al., 2015)	Detection	Generic	115.00	32.53
S-RPN (Lin et al., 2017)	Detection	Generic	24.32	37.62
RetinaNet (Hsieh et al., 2017)	Detection	Generic	16.62	22.30
LPN (Lin et al., 2017)	Detection	Generic	23.80	36.79
One look (Mundhenk et al., 2016)	Detection	Specific	59.46	66.84
IEP count (Stahl et al., 2018)	Detection	Specific	51.83	-
PDEM (Goldman et al., 2019)	Detection	Specific	6.77	8.52
GMN (Lu et al., 2018)	Regression	Generic	7.48	9.90
FamNet+ (Ranjan et al., 2021)	Regression	Generic	18.19	33.66
BMNet+ (Shi et al., 2022)	Regression	Generic	5.76	7.83
SAFEcount (You et al., 2023)	Regression	Generic	5.33	7.04
HMFENet (our)	Regression	Generic	5.17	7.03

### 4.6. Discussion

In the prior section, we compared our method to the existing methods. In Table 2, we have observed that our method sets a new state-of-art on the standard dataset FSC-147. Figure 3 shows that our model still performs quite well in the face of dense objects and a large number of overlapping occlusion phenomena. The boundary of the density map is clear and close to the real density map.

We use local self-attention (LSA) to optimize the tensor of the query image, which distinguishes the background from the target object based on the context information. The exemplar feature is a key link in FSC, but due to light changes, shape, and even color differences, there may be large changes between exemplars. To improve the common feature of exemplars and reduce the unimportant characteristics, we propose an exemplar feature aggregation module (EFA) to enhance the commonality of exemplar features. The Hough matching is the important part of our study, which builds accurate matching in the face of severely deformed and occluded objects. The ablation studies show our modules are useful, and the results get improved on the FSC-147 dataset.

Although our approach is focused on few-shot object counting, related experiments are also performed on class-specific object counting. Experiments show that our method still outperforms other methods on the CARPK dataset.

## 5. Conclusion

For FSC, the key point is how to shorten the distance between the exemplars and objects of the same class in the query image and push away the objects of different classes. Establishing reliable similarity maps is an important part. First, we refine the query tensor by local self-attention and enhance the commonality of exemplar feature tensors by exemplar feature aggregation module, which significantly improves the robustness of counting accuracy. Then, we apply the Hough matching module to replace the traditional convolution. And the experiment results show that the performance of matching has been improved. Finally, we use a feature enhancement module to integrate the exemplar features into query features, which can pull the features between the exemplar and target instances closer and get clear borders within dense objects. Experiment results demonstrate that our HMFENet reaches a new sate-of-art on the standard dataset FSC-147 and performs best on the class-specific dataset CAPRK.

## Data availability statement

Publicly available datasets were analyzed in this study. This data can be found here: https://github.com/cvlab-stonybrook/LearningToCountEverything.

## Author contributions

ZH proposed the HMFENet and wrote the manuscript. DZ conducted the literature survey and provided method guidance. HW analyzed the experiment data and revised the manuscript. All authors contributed to the article and approved the submitted version.
