# Corrigendum: Accurate few-shot object counting with Hough matching feature enhancement

**DOI:** 10.3389/fncom.2023.1232762

**Published:** 2023-06-20

**Authors:** Zhiquan He, Donghong Zheng, Hengyou Wang

**Affiliations:** ^1^Guangdong Key Laboratory of Intelligent Information Processing, Shenzhen, China; ^2^Guangdong Multimedia Information Service Engineering Technology Research Center, Shenzhen University, Shenzhen, China; ^3^School of Science, Beijing University of Civil Engineering and Architecture, Beijing, China

**Keywords:** few-shot, object counting, Hough matching, feature enhancement, exemplar feature aggregation, self-attention

In the published article, there was an error in the **Funding** statement.

Original text: This work was supported by the National Natural Science Foundation of China under Grants 61971290.

The Shenzhen City project support was missed by mistake. The correct Funding statement appears below.

This work was supported by the National Natural Science Foundation of China under grant 61971290, and the Shenzhen Stability Support General Project (Category A) 20200826104014001.

The authors apologize for this error and state that this does not change the scientific conclusions of the article in any way. The original article has been updated.

